# Characterization of Serum Phospholipase A_2_ Activity in Three Diverse Species of West African Crocodiles

**DOI:** 10.1155/2011/925012

**Published:** 2011-10-29

**Authors:** Mark Merchant, Kate Juneau, Jared Gemillion, Rodolfo Falconi, Aaron Doucet, Matthew H. Shirley

**Affiliations:** ^1^Department of Chemistry, McNeese State University, 450 Beauregard, Kirkman Hall 221A, Lake Charles, LA 70609, USA; ^2^Department of Wildlife Ecology & Conservation, University of Florida, Gainesville, FL, USA

## Abstract

Secretory phospholipase A_2_, an enzyme that exhibits substantial immunological activity, was measured in the serum of three species of diverse West African crocodiles. Incubation of different volumes of crocodile serum with bacteria labeled with a fluorescent fatty acid in the *sn*-2 position of membrane lipids resulted in a volume-dependent liberation of fluorescent probe. Serum from the Nile crocodile (*Crocodylus niloticus*) exhibited slightly higher activity than that of the slender-snouted crocodile (*Mecistops cataphractus*) and the African dwarf crocodile (*Osteolaemus tetraspis*). Product formation was inhibited by BPB, a specific PLA_2_ inhibitor, confirming that the activity was a direct result of the presence of serum PLA_2_. Kinetic analysis showed that *C. niloticus* serum produced product more rapidly than *M. cataphractus* or *O. tetraspis*. Serum from all three species exhibited temperature-dependent PLA_2_ activities but with slightly different thermal profiles. All three crocodilian species showed high levels of activity against eight different species of bacteria.

## 1. Introduction

The phospholipase A_2_ (PLA_2_) family is a diverse set of enzymes represented by 15 different groups that include five specific types: cytosolic (cPLA_2_), secreted (sPLA_2_), Ca^2+^-independent (iPLA_2_), platelet-activating factor acetylhydrolases (PAF-AH), and the lysosomal PLA_2_s [1, 2] These enzymes are characterized by their ability to cleave fatty acids from the *sn*-2 position of membrane lipids. Some of the cPLA_2_s have long been recognized for their roles in the biosynthesis of eicosanoids, paracrine hormones that mediate a broad spectrum of physiological processes [3, 4] In general, the PLA_2_ family of enzymes can be divided into two main groups: intracellular and extracellular (secreted) PLA_2_ forms. 

Higher eukaryotes must continuously defend against infection by potentially infectious microbes. Innate immunity is the first line of defense against infection [5]. It is typically nonspecific in its action, thus allowing for broad-spectrum activity. Secretory PLA_2_ (sPLA_2_) has been shown to exhibit substantial innate immune activity [6, 7]. PLA_2_ is an enzyme that catalyzes the hydrolysis of fatty acids from the *sn*-2 position of membrane lipids [6]. The sPLA_2_ isoform was first described by Vadas et al. [3, 4], and was later described as having antibacterial properties [8–10]. The antibacterial activity of sPLA_2_ has been largely attributed to the cationic nature of the enzyme, thus allowing for interaction with, and disruption of, microbial membranes [11]. Other studies have shown that the antimicrobial effects of sPLA_2_ can be modulated by antimicrobial peptides [12]. The presence of this enzyme in inflammatory fluids [6], human tears [13], intestinal Paneth cells [14], and macrophages [15] is consistent with its role as an important component of innate immunity. 

Three diverse crocodilian species, each belonging to a different genus, can be found sympatrically in western Central Africa. These three species vary greatly with respect to morphology and general ecology. The slender-snouted crocodile (*Mecistops cataphractus*) and the African dwarf crocodile (*Osteolaemus tetraspis*) are considered the most poorly understood crocodilians in the world, with respect to nesting, feeding, and general ecology [16]. *M. cataphractus *is a medium-bodied species that inhabits forested rivers and other wetlands in Central and West Africa. As its name suggests, the slender-snouted crocodile is a longirostrine species adapted for the speed necessary of a highly aquatic, piscivorous lifestyle [17–19]. *O*.* tetraspis *is a small, stocky crocodilian that can be found in a broad spectrum of habitats including inundated forests, mangrove swamps, and papyrus marshes. The Nile crocodile (*Crocodylus niloticus*) is among the most studied of all crocodilian species. The current distribution of *C. niloticus *in the study area is limited to coastal lagoons surrounded by savannah-forest mosaic, including frequent use of the marine environment for dispersal and seasonal movements [20, M. J. Eaton and M. H. Shirley pers. obs.]. Previous studies have shown that these three divergent species exhibit different immune activities toward different bacterial species [21]. 

Considering their protected status, little is known about crocodilian physiology and biochemistry. However, during the past five years, several studies have focused on the innate immunity of crocodilians [21–26]. Nevalainen et al. [27] first showed that crocodilians (*Crocodylus porosus *and *Crocodylus siamensis*) express serum PLA_2_ activity. Later, Merchant et al. showed that the American alligator (*Alligator mississippiensis*) [28] exhibited serum PLA_2_ activity. This study was undertaken to determine the serum PLA_2_ activity of three diverse species of Centeral African crocodiles.

## 2. Materials and Methods

### 2.1. Chemicals and Biochemicals

4,4-difluoro-5,7-dimethyl-4-bora-3a,4a-diaza-s-indacene-3-hexadecanoic acid (BODIPY FL C_16_) was purchased from Invitrogen (Carlsbad, CA, USA). Ethylene glycol tetraacetate (EGTA), *p*-bromophenacyl bromide (BPB), CaCl_2_, nutrient broth, sodium hydroxide, and tris HCl were purchased from Sigma Chemical Co. (St. Louis, MO, USA).

### 2.2. Treatment of Animals

Crocodiles were captured from the N'Dougou Lagoon, Bongo River, and Nyanga River areas of the Gamba Complex region in Gabon, Africa. Animals were captured using standard crocodilian capture techniques, including by hand, tongs, locking snare, or darting [29]. Blood was collected from crocodiles via the spinal vein [30, 31]. Five mL of whole blood were collected from animals that were less than one meter in total length, and larger volumes were collected from larger crocodilians, commensurate with length and body condition. All animals were released at the site of capture. All of the activities were approved by McNeese State University and University of Florida Animal Care and Use Committees. The serum samples for each species were pooled (14–30 per species) such that average enzymatic activities could be obtained for each species.

### 2.3. Labeling of Bacteria

One-mL cultures of *E. coli *bacteria were grown overnight at 37°C in nutrient broth. These cultures were used to inoculate one liter cultures. These cultures were incubated for 24 hours in the presence of 1 mg of BODIPY FL C_16_ (dissolved in one mL of DMSO). The bacteria were centrifuged at 8000 ×g for 15 min., the cultures were decanted, and the bacteria were resuspended in 30 mL of sterile isotonic saline. The bacteria were again centrifuged 8000 ×g for 15 min. to remove unincorporated BODIPY, the bacterial pellet was resuspended in 30 mL of sterile isotonic saline and frozen at −20°C until ready for use.

### 2.4. PLA_2_ Assay

The method used for the determination of crocodile serum PLA_2_ enzyme activity has been recently described [28]. Crocodile serum was incubated with 250 *μ*L of assay buffer (1 mM Ca^2+^ in 100 mM tris-HCl, pH 7.4) and 100 *μ*L of BODIPY-labeled bacteria. The balance of the 750 *μ*L reaction consisted of isotonic saline. For the determination of the effects of serum volume on PLA_2_ activity, different amounts of crocodile serum were incubated with 50 *μ*L of BODIPY-labeled *E. coli *bacteria in assay buffer for 30 min ambient temperature. The reactions were terminated by the addition of 750 mL of stop buffer (100 mM Tris-HCl, pH 7.4 with 15 mM EDTA) and were then centrifuged at 16,000 ×g to pellet the labeled bacteria, and 650 *μ*L of each reaction were removed to a one mL plastic cuvette. The fluorescent intensity of each reaction was measured at an excitation *λ* of 500 nm and an emission *λ* of 510 nm (excitation and emission slit widths = 1 nm) in a Horiba Jobin Yvon Fluoromax-4 fluorimeter. The same procedure was followed to determine the effects of time, temperature, and inhibitors on crocodile PLA_2_ activities.

### 2.5. Statistics and Controls

The fluorescent intensity of each sample was compared to a standard curve of pure product to determine the nmols of product formed. The fluorescent intensities of each sample were corrected for background fluorescence by subtraction of a reagent blank in the absence of serum. Each data point represents the means ± standard deviation for four independent determinations. The results obtained from each experiment were subjected to analysis of variance using Scheffe's post hoc comparisons [32].

## 3. Results 

**Figure 1:**
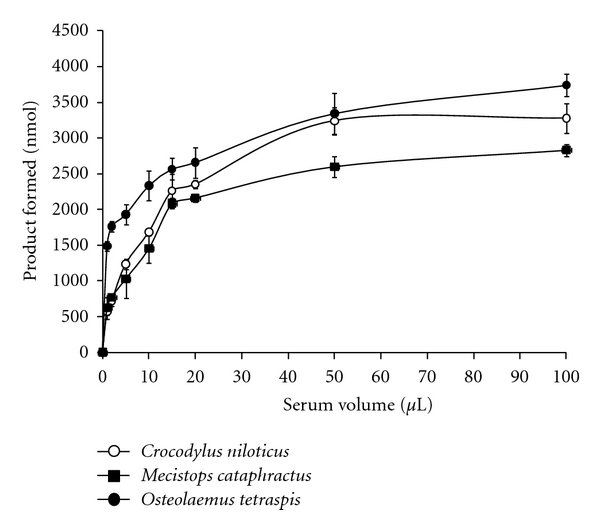
Serum volume-dependent PLA_2_ activity in *Osteolaemus tetraspis*, *Mecistops cataphractus*, and *Crocodylus niloticus*. The results represent the means ± standard deviations of four independent determinations.

The results of the serum volume-dependent PLA_2_ activity for *O. tetraspis*, *M. cataphractus*, and *C. niloticus *are displayed in Figure 1. Substantial PLA_2_ activity (*P* < 0.01) was measured in all three crocodilian species with the use of only one *μ*L of serum. The PLA_2_ activities measured for *Osteolaemus *and *Mecistops *were similar to each other throughout the entire range of serum volumes. However, the PLA_2_ activity for *C. niloticus *was substantially higher than that of the other two species at serum volumes of 20 *μ*L and higher (*P* < 0.01). At low serum volumes, the activity increased rapidly and then increased more slowly at volumes greater than 20 *μ*L. 

**Figure 2:**
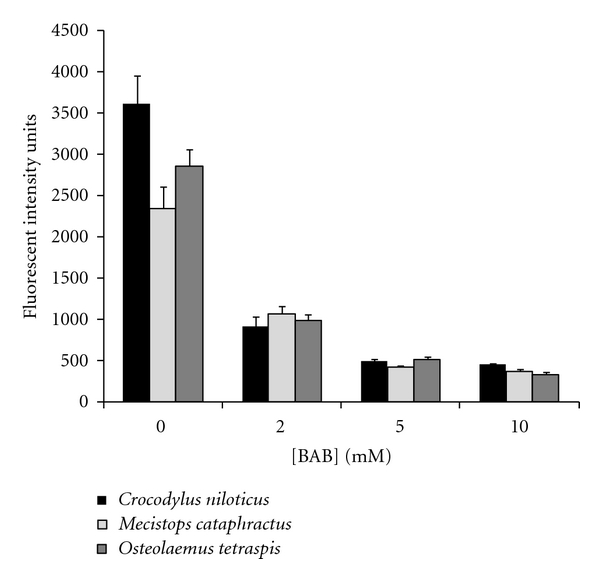
The effects of p-bromophenacyl bromide, a specific PLA_2_ inhibitor, on the serum PLA_2_ activity of *Osteolaemus tetraspis*, *Mecistops cataphractus*, and *Crocodylus niloticus*. The results represent the means ± standard deviations of four independent determinations.

The effect of BPB, a specific PLA_2_ enzyme inhibitor, on the PLA_2_ activity of crocodile serum is illustrated in Figure 2. Incubation of *O. tetraspis*, *M. cataphractus*, and *C. niloticus *serum in the absence of inhibitor resulted in 2860 ± 346, 2342 ± 263, and 3919 ± 204 nmol, respectively. However, incubation of serum from *O. tetraspis*, *M. cataphractus *and *C. niloticus *serum with 2 mM BPB resulted in a 65%, 55%, and 75% inhibition of PLA_2_ activity, respectively. An increase in the BPB concentration to 5 and 10 mM only increased the enzyme inhibition slightly. 

**Figure 3:**
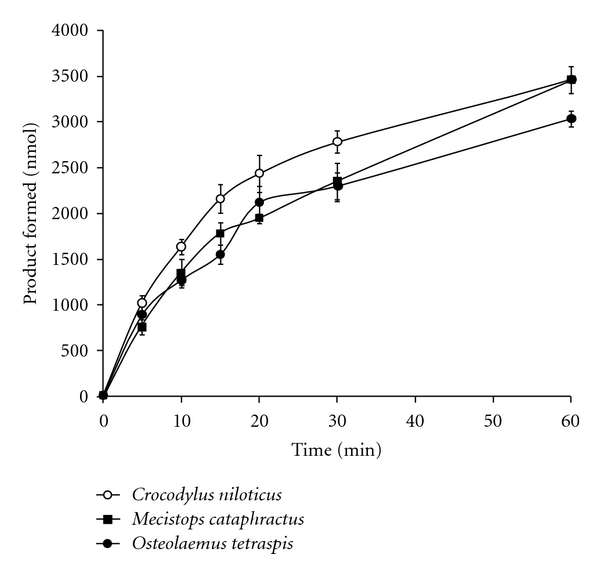
The results represent the means ± standard deviations of four independent determinations.

Figure 3 shows the time-dependent PLA_2_-dependent hydrolysis of BODIPY by three species of Central African crocodiles. Accumulation of product occurred rapidly during the first 20 min. of incubation with substrate, and then decreased after 20 min. Serum from *C. niloticus *exhibited a higher rate of product formation than *O. tetraspis *and *M. cataphractus *serum. Enzyme activity was detected as early as one minute after incubation of substrate with serum from all three crocodilian species. Within 10 minutes of incubation with substrate, the *C. niloticus *serum exhibited statistically higher activity (*P* < 0.01) than that from *O. tetraspis *or *M. cataphractus*. 

**Figure 4:**
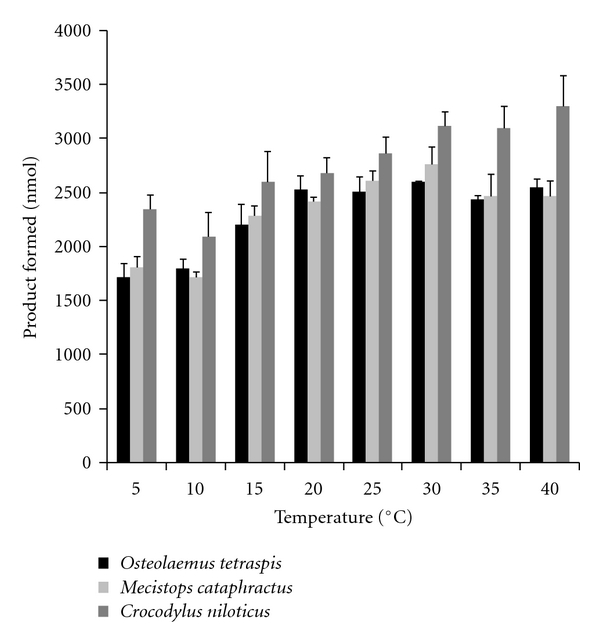
Serum PLA_2_ activity of *Osteolaemus tetraspis*, *Mecistops cataphractus*, and *Crocodylus niloticus *at different temperatures. The results represent the means ± standard deviations of four independent determinations.

Figure 4 illustrates the temperature-dependent PLA_2_ serum exhibited by the three African crocodile species. The thermal profile for *C. niloticus *serum PLA_2_ showed an increase in activity throughout the temperature range. However, the accumulation of product at the highest temperature (3299 nmol, 40°C) was only 41% higher than that recorded at the lowest temperature (2342 nmol, 5°C). In contrast, *M. cataphractus *serum PLA_2_ activity increased from 5–30°C, where it peaked and then decreased at 35 and 40°C. The thermal profile for *O. tetraspis *serum PLA_2_ increased steadily from 5–20°C, where it remained relatively constant from 20–40°C. 

**Figure 5:**
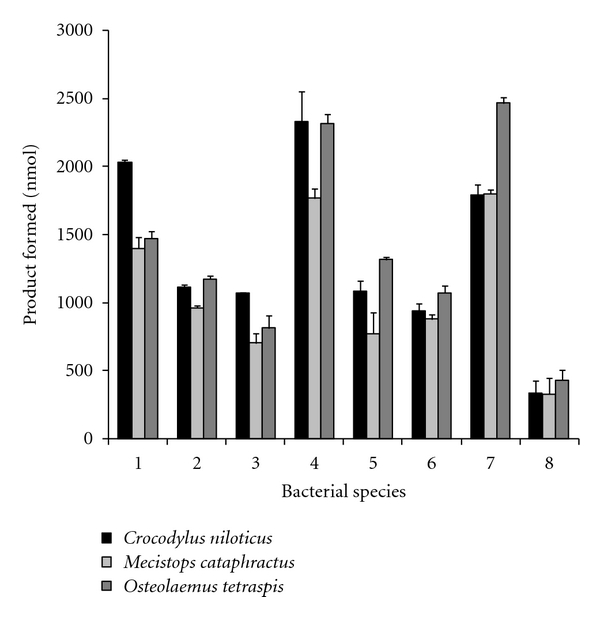
Phospholipase A_2_ activity of *Osteolaemus tetraspis*, *Mecistops cataphractus*, and *Crocodylus niloticus *serum against different bacterial species. The results represent the means ± standard deviations of four independent determinations. 1; *Enterobacter cloacae*, 2; *Shigella flexneri*, 3; *Salmonella typhi*, 4; *Klebsiella oxytoca*, 5; *Providencia stuartii*, 6; *Streptococcus pyrogens*, 7; *Streptococcus faecalis*, 8; *Staphylococcus aureus*.

 The effects of crocodile serum PLA_2_ on cleavage of fatty acids from eight different bacterial species are shown in Figure 5. In general, the serum PLA_2_ from all three species of crocodilians showed high activity against *Enterobacter cloacae*, *Klebsiella oxytoca, *and *Streptococcus faecalis*, while the lowest activities were recorded against *Staphylococcus aureus*. Serum from *C. niloticus *was more active (*P* < 0.01) against *Enterobacter cloacae *and *Salmonella typhi *than that from the other two crocodilian species. However, serum from *O. tetraspis *exhibited higher activity against *Providencia stuartii *and *Streptococcus pyrogens*, relative to serum from the other two African crocodilians.

## 4. Discussion

Lipases, which are subsets of esterase enzymes, constitute a large group of enzymes that are capable of hydrolysis of lipid ester bonds [33]. Lipases are utilized for a broad variety of biological functions, including signal transduction, synthesis of hormones, cholesterol metabolism, serum lipoprotein balance, innate immunity, and so forth. Because of the possibility that the hydrolysis activities observed were due to enzymes other than PLA_2_, the effects of BPB, a specific inhibitor of PLA_2_ activity [34], were observed on the cleavage of fluorescently labeled fatty acid from the surface of *E. coli *bacteria (Figure 2). The data clearly show that the BPB inhibits fluorescent product formation in the serum of all three species in a concentration-dependent manner. These data indicate that the lipolysis activities measured are likely due to the presence of PLA_2_ in crocodilian serum. It is worthy to note that another enzyme with PLA_2_-like activity, PAF-acetylhydrolase, is not inhibited by BPB. However, it has also been shown that this enzyme does not require Ca^2+^ and thus would not be inhibited by PLA_2_ [35]. Therefore, any PAF-acetylhydrolase activity in the serum would have been subtracted as background activity due to the fact that the reaction would not have been stopped by the addition of Stop Buffer, which contained EDTA. 

All three West African crocodilian species exhibited high levels of serum PLA_2_, relative to other crocodilian species [28]. However, *C. niloticus *showed overall higher PLA_2_ activity than *O. tetraspis *and *M. cataphractus *with respect to the volume of serum (Figure 1), more rapid accumulation of product in kinetic experiments (Figure 3), and higher activity across a broad range of temperatures (Figure 4). This could potentially be a result of the more aggressive lifestyle of *C. niloticus*, relative to the other two species. *C. niloticus *is a highly social species with well-established breeding and dominance hierarchies, through aggressive maintenance of territories, which often results in extensive injury [36–38]. This might lead to an increased rate of injury during feeding and/or territory defense, compared to *Osteolaemus *and *Mecistops*, and thus the need for more potent innate immunity. 

The physiology, biochemistry, and metabolic rates of ectothermic vertebrates are largely dependent on the temperatures of their environments [39]. Serum enzyme activities for crocodilians have been shown to be extremely temperature dependent [40]. For instance, the serum complement system in *Alligator mississippiensis *[22], *Crocodylus porosus *and *Crocodylus johnstonii *[41], *Caiman latirostris *[42], and *Crocodylus acutus *[43] are all temperature dependent. It is interesting to note that serum PLA_2_ activity for both *O. tetraspis *and *M. cataphractus *increased from 5–20°C (Figure 4) and then remained relatively constant or decreased slightly at higher temperatures (35 and 40°C). However, the PLA_2_ activity for *C. niloticus *increased throughout the entire range of temperatures observed. This might be a result of the temperature differences in the habitat selection for the three species in this study. 

While all three crocodilians in this study can be found syntopically, *C. niloticus*, *M. cataphractus*, and *O. tetraspis *exhibit niche partitioning through differential foraging, nesting ecology, habitat preference, and morphology. *O. tetraspis *is usually found in flooded forests and other small wetlands/waterways under canopy cover in heavily forested areas [18, 44, 45]. In addition, this species makes extensive use of underground burrows [16, 18, 19]. Similarly, *M. cataphractus *typically inhabits rivers, flooded forests, and swampy wetlands in heavily forested areas [17, 18, 44]. In contrast, ideal *C. niloticus *habitat is more open and includes exposed water and ample open shoreline for basking and nesting [46, 47]. The habitat chosen by *C. niloticus *is more prone to higher temperatures due to the lack of shaded areas, while the forested habitats most often selected by *O. tetraspis *and *M. cataphractus *would tend to be more shaded and thus lower in temperature. Therefore, observed differences in temperature profiles and optima for the PLA_2_ activities in these species may reflect microclimatic variation in preferred habitats. 

This observed pattern may be supported by the enzyme thermodynamic profiles seen in crocodilians that inhabit more temperate versus tropical latitudes. Temperate species exhibit lower temperature optima than more tropical species [22]. Although serum PLA_2_ activities for both *O. tetraspis *and *M. cataphractus *were lower than *C. niloticus*, they were both approximately 30% higher than that measured in *Alligator mississippiensis *[28]. *A. mississippiensis *is one of the most temperate crocodilians, even commonly found in regions with frequent winter freeze events [47]. Though, while the absolute amount of product formation was higher in the African crocodile species, the rate of product formation was lower than that for *A. mississippiensis *(Figure 3) [28]. It may be that species occurring in colder climates are adapted to quicker immune response to counteract the immune suppression caused by lower core body temperatures. Lower absolute product formation with high initial formation rates are mirrored in other temperately distributed species (e.g., *Caiman latirostris*) [42]. 

The species relationships in enzymatic response observed in this study could also represent underlying evolutionary relationships in innate immunity [48, 49]. Recent studies in molecular phylogenetics have confirmed a sister taxa relationship between *Osteolaemus *and *Mecistops*, which form a clade sister to the true crocodiles of the genus *Crocodylus *[50–54]. PLA_2_ temperature profiles and optima for *Crocodylus acutus *mirrored that of *C. niloticus*, while *Osteolaemus *and *Mecistops *are intermediate from that seen in alligatorids (*Alligator mississippiensis* [28], *Caiman latirostris *and *Caiman yacare *(Merchant, unpublished observations)). Molecular and fossil dating estimates confirm that the family Crocodylidae, and in particular the genus *Crocodylus*, is the newest and most derived member of the Crocodylia, while the Alligatoridae is the oldest and most ancestral clade [50, 52–56]. These results suggest an evolutionary basis for enzyme activity which supports results from previous studies using immunological data to inform phylogenetics of the Crocodylia [57, 58]. 

Serum from all three West African crocodilians exhibited substantial PLA_2_ activities toward all eight bacterial species tested. This is not surprising, considering that PLA_2_ is a nonspecific enzyme with activity that is not dependent on the presence of particular antigens. The activities of PLA_2_ enzymes depend on the content (lipid content, fluidity, charge density, etc.) of the membrane [59–61]. It is interesting to note that all three crocodiles exhibited PLA_2_ activities against all eight bacteria, which included both Gram-positive (*n* = 3) and Gram-negative (*n* = 5) species. These results indicate that crocodilian PLA_2_ is potentially effective against a broad spectrum of diverse bacteria. 

This study has provided evidence of differential PLA_2_ expression in three species of diverse African crocodilian species. The PLA_2_ activity was serum volume-, time-, and temperature-dependent. The PLA_2_ activities were broad spectrum in character, affecting eight different bacterial species and also inhibited by BPB. Although this investigation did not include *in vivo *studies, it is reasonable to expect that serum PLA_2_ constitutes an important component of crocodilian innate immunity.
